# Enhanced Chemosensory Detection of Negative Emotions in Congenital Blindness

**DOI:** 10.1155/2015/469750

**Published:** 2015-03-23

**Authors:** Katrine D. Iversen, Maurice Ptito, Per Møller, Ron Kupers

**Affiliations:** ^1^BrainLab, Department of Neuroscience & Pharmacology, Panum Institute, Faculty of Health & Medical Sciences, University of Copenhagen, 2200 Copenhagen, Denmark; ^2^Laboratory of Neuropsychiatry, Psychiatric Centre Copenhagen, 2200 Copenhagen, Denmark; ^3^School of Optometry, Université de Montréal, Montréal, QC, Canada H3T 1P1; ^4^Department of Food Science, University of Copenhagen, 1958 Frederiksberg, Denmark

## Abstract

It is generally acknowledged that congenitally blind individuals develop superior sensory abilities in order to compensate for their lack of vision. Substantial research has been done on somatosensory and auditory sensory information processing of the blind. However, relatively little information is available about compensatory plasticity in the olfactory domain. Although previous studies indicate that blind individuals have superior olfactory abilities, no studies so far have investigated their sense of smell in relation to social and affective communication. The current study compares congenitally blind and normal sighted individuals in their ability to discriminate and identify emotions from body odours. A group of 14 congenitally blind and 14 age- and sex-matched sighted control subjects participated in the study. We compared participants' abilities to detect and identify by smelling sweat from donors who had been watching excerpts from emotional movies showing amusement, fear, disgust, or sexual arousal. Our results show that congenitally blind subjects outperformed sighted controls in identifying fear from male donors. In addition, there was a strong tendency that blind individuals were also better in detecting disgust. Our findings reveal that congenitally blind individuals are better at identifying ecologically important emotions and provide new insights into the mechanisms of social and affective communication in blindness.

## 1. Introduction

Vision plays an important role in social and affective communication. This is well illustrated by the fact that nonhuman and human primates have brain areas such as the superior temporal sulcus and the fusiform area that respond preferentially to social stimuli such as faces and body gestures [1]. Indeed, when assessing the emotional state of a fellow human, we rely heavily on visual cues of facial expressions [2, 3] and on whole body gestures [4]. These visual cues provide us with explicit and salient information about the intentions of a person, about his/her mood (happy, angry, or depressed), whether the person is trustworthy or not, and so forth. Recognition of the identity, intentions, and emotional state of our fellow humans is hence crucial for survival. This raises the question of how blind people assess the emotions in others and how they judge their intentions and trustworthiness. We have previously demonstrated that, compared to sighted controls, congenitally blind individuals have a lower odour identification threshold and are more aware of odours in their environment, especially social odours [5]. This suggests that blind subjects are more sensitive to olfactory input and pay more attention to body odours in social communication.

Odour perception is one of the most basic channels of social communication. There is ample evidence that animals use smells to mark their territory, initiate sexual encounters, differentiate among individuals, and identify the emotional state of the other [6–8]. Even though the olfactory system in humans is not very different from that of other mammalians [7], olfaction plays a less prominent role in human behaviours as compared to vision and audition [9]. However, previous studies in humans have shown that natural body odours carry biological salience [10] and influence social interactions [11, 12]. Given the facts that the olfactory system is highly plastic [13] and that blind individuals show compensatory plasticity for various forms of sensory processing [14], we hypothesized that they would be better than sighted controls in detecting emotions of a fellow human. The present study probes this issue by assessing the ability to discriminate and identify chemosensory emotional cues in congenitally blinds and sighted controls.

## 2. Materials and Methods

### 2.1. Participants

A group of 14 congenitally blind (7 females, mean age: 44 ± 3 years) and 14 sighted controls, age- and sex-matched (6 females, mean age: 41 ± 3 years), were enrolled in the study. We recruited only heterosexuals since sexual orientation has been shown to influence perception and cerebral responses to body odours [15–17]. All participants gave their written informed consent prior to participation and the local ethics committee of the county of Copenhagen and Frederiksberg approved the study. Demographic data and causes of blindness are summarized in Table 1. None of the participants reported any current psychiatric diseases, nasal deformities, fractures, obstruction, past repeated exposure to vaporous chemicals, consumption of inhaled nonmedical drugs, neurological diseases, nor respiratory problems.

### 2.2. Materials and Procedure

We controlled for possible confounding variables such as general olfactory impairment and odour awareness. Odour identification ability was assessed using the Monex 40 Sniffin' Sticks battery [18], consisting of 40 Sniffin' Sticks that assess one's ability to identify common household smells using a multiple-choice procedure. Participants who scored lower than 20 were excluded from further participation. The main study consisted of a sweat collection and a sweat judgment procedure.

*Phase 1: Sweat Collection*. A group of 30 (15 females) young healthy participants (mean age: 25 ± 6 years) were recruited as sweat donors in the experiment. None of them participated in the ensuing sweat judgment task. Participants refrained from using deodorants or antiperspirant-scented products and were asked to use scent-free shampoo and soap that was provided by the experimenter from two days prior to the sweat collection sessions until the end of the sessions. Sweat donors also were asked to avoid consuming odorous foods such as garlic, onion, asparagus, blue cheese, and strong spices. The night prior to the experiment, the sweat donors were asked to sleep in a fresh t-shirt washed in scent-free soap (provided by the experimenter) in order to avoid contamination by odours from their bed sheets. On the day of the sweat collection session, participants wore a new t-shirt (provided by the experimenter) over their skin to prevent odour contamination by their regular clothes. During the sweat collections session, donors kept a cotton pad under their armpits while watching a 20 min video intended to produce one of the following emotions: amusement, fear, disgust, or sexual arousal. The participants were seated individually in a medium sized room with controlled temperature, where the film was presented on a large TV screen. The emotions were induced with film excerpts tailored to the donor. Prior to the video session, each donor had completed an extended questionnaire about his or her film preferences, encompassing questions such as what type of movie they normally have the strongest reaction to and writing down examples of movies they previously had a strong emotional reaction to. Based on their answers, participants were chosen to watch a movie with a particular emotional content that was in accordance with their personal preferences. During watching the movie clips, hemodynamic responses (heart rate) were recorded noninvasively using Finapres NOVA. Prior to the start of the movie, participants rated baseline levels of each of the four different emotions, using a 7-point Likert scale. Thereafter, we took a 5-minute baseline measurement of heart rate. At the end of the 20 min video clip, participants rated how angry, fearful, happy, disgusted, sad, and sexually aroused they felt during the video. Further analyses pertaining to mood induction were based on these selected videos/sweat samples. Valid hemodynamic data were recorded from 29 participants.

Once collected, sweat pads were cut into four even pieces and stored in separate jars, coded by an individual not involved in the study. Each jar contained four pieces of sweat pads from four different donors of the same sex who had been watching the same type of emotional movie, for example, fear. The sweat pads were then kept at −20 to −26°C until subsequent testing [19]. Unused cotton pads served as control stimuli because other nonemotional bodily secretions (e.g., sweat from a workout) can potentially contain chemosignals [11]. The control stimuli were stored in the same manner as the emotional sweat samples. In the sweat evaluation phase, we used two sets of jars, one from each gender. Each set contained 5 jars with sweat samples of amusement, fearful, sexual arousal, and disgust and control pads, respectively.

**Figure 1:**
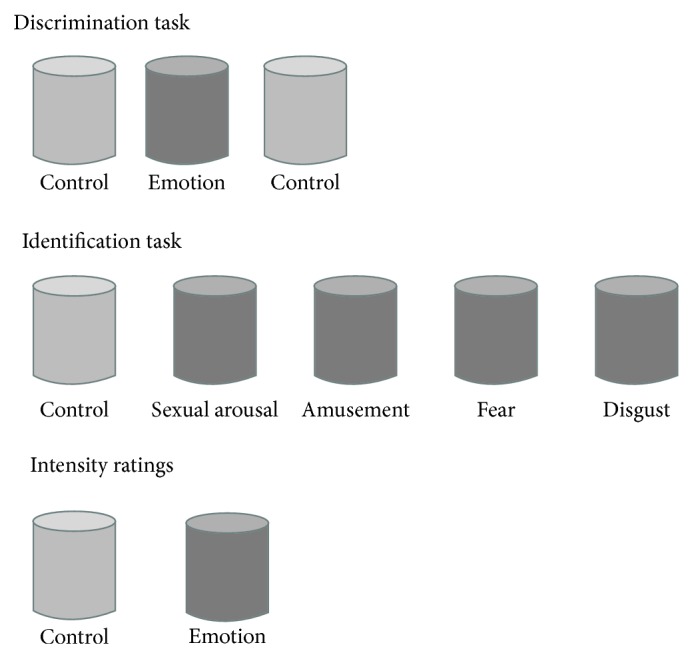
Graphical representation of the different tasks. The participants started with a detection task, followed by an identification task. Lastly, they had to rate the intensity of each emotion and the controls. The bottles with light shades represent the controls, whereas the darker shaded bottles represent the emotions.

*Phase 2: Sweat Judgment*. Sweat samples were presented in a double-blind manner. Participants judged the sweat samples first in a discrimination task, then in an identification task, and finally in an intensity rating task, schematically represented in Figure 1. All participants, including the blind, were blindfolded during data acquisition. In the odour discrimination task, participants were presented with three jars and they were asked to take an inhalation through the nose and exhale through the mouth and select the smell that was different from the other two. Two of the jars contained crumbled pads and the third contained an emotional (disgust, fear, sexual, or amusement) sweat pad. The task was repeated 4 times for each emotion. Since sweat samples were separated by gender, we had 32 trials in total (4 emotions × 4 repetitions × 2 genders), presented in a pseudorandomized fashion, with a 20–30 s interval between the trials. In the odour identification task, participants were presented with all 5 jars and they were asked to identify the jar containing the smell of one particular emotion (amusement, fear, sexual arousal, or disgust). This task was repeated twice for each emotion and for each gender donor, resulting in 16 trials presented in a pseudorandomized fashion. The trial ended with an intensity rating, where participants had to rate the intensity of each odour on a 7-point Likert scale (1 = not at all, 7 = very much so).

### 2.3. Statistical Analyses

We performed Wilcoxon rank-sum tests to assess if congenitally blind individuals were better at detecting chemosensory emotional cues. The same procedure was applied to examine the effects of vision on the identification of the emotional contents of the sweat samples. We used a repeated-measure ANOVA to assess whether the intensity and pleasantness of the sweat samples were rated differently for the different emotions. Levels of *P* < 0.05 were considered as statistically significant. Data are presented as mean ± SEM.

## 3. Results

### 3.1. General Odour Perception

None of the participants had to be excluded for reasons of anosmia or hyposmia. Blind and sighted participants did not differ in their ability to identify common household smells; mean scores on the Monex-40 were 27 ± 5 and 29 ± 4 for blind and sighted, respectively (*t*(26) = 1417, *P* = 0.168). Normative data for the Monex is 31.7 ± 2.95, as measured in a North-American population.

### 3.2. Mood Induction

**Figure 2:**
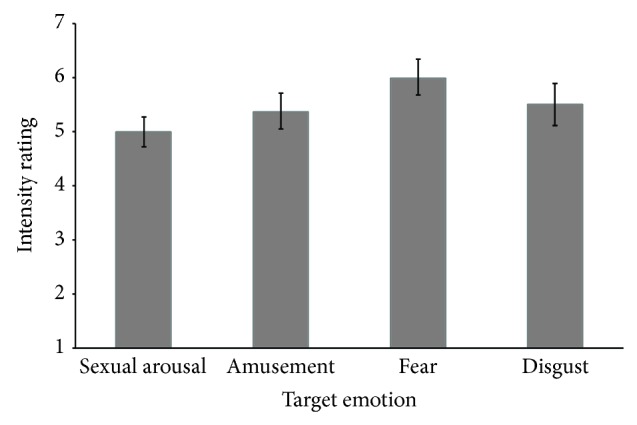
Emotional responses to the movie clips. Mean of self-reported emotions on a 7-point Likert scale while participants watched amusing, disgusting, fearful, and sexual arousing video excerpts. Data present mean ± SD.

Figure 2 shows that watching the movie clips successfully induced the targeted emotions in the odour donors. The baseline values before the start of the movie for fear, sexual arousal, disgust, and amusement were 1.38 ± 0.18, 1.5 ± 0.32, 1 ± 0.0, and 4.3 ± 0.5, respectively. The self-reported experienced emotions after watching the films rose to scores between 5 and 6 on the 7-point Likert scale. Although the change in amusement rating was only one point, all participants scored higher on this emotion during the film.

Participants' hemodynamic responses during the movie clips confirmed their subjective reports. Indeed, heart rate increased significantly during movies showing fear, disgust, and amusement, whereas there was a strong trend for sexual arousal (Table 2).

### 3.3. Emotional Odour Discrimination

**Figure 3:**
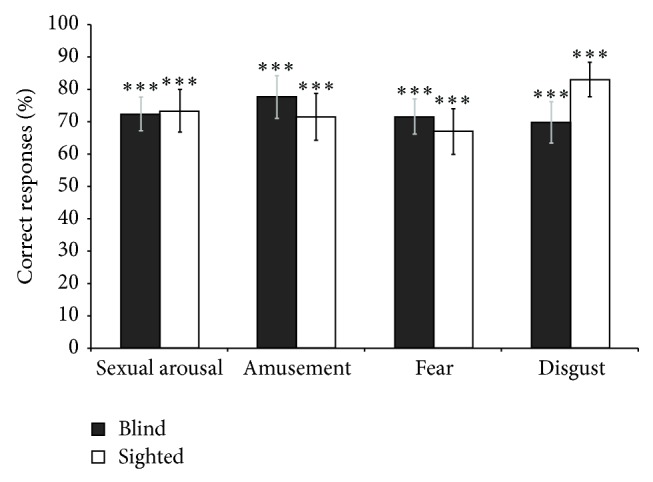
Results of the odour discrimination task. Both groups were significantly above chance level (33%) in detecting emotional chemosensory pads from control pads. Blind participants did not differ from sighted controls. Data present mean ± SD. ^***^ *P* < 0.001.

As shown in Figure 3, both groups were overall significantly above chance level in discriminating sweat pads from control pads (*P* < 0.001). Mean accuracy of the blind for detecting disgust, fear, sexual arousal, and amusement was 72 ± 5%, 78 ± 6%, 70 ± 6%, and 71 ± 5%, respectively, compared to 73 ± 7%, 71 ± 7%, 83 ± 5%, and 67 ± 7% for the sighted (chance level = 33%). The above chance level performance for chemosensory emotional discrimination in both groups is unlikely due to the fact that the emotional sweat samples were perceptually more intense than the control samples since there were no significant differences in intensity between the emotional sweat and the control samples (*P* values > 0.47). It is also unlikely that the above chance level performance is due to differences in the pleasantness ratings of the emotional and control samples as these did not differ (*P* values > 0.33).

### 3.4. Enhanced Chemosensory Identification

**Figure 4:**
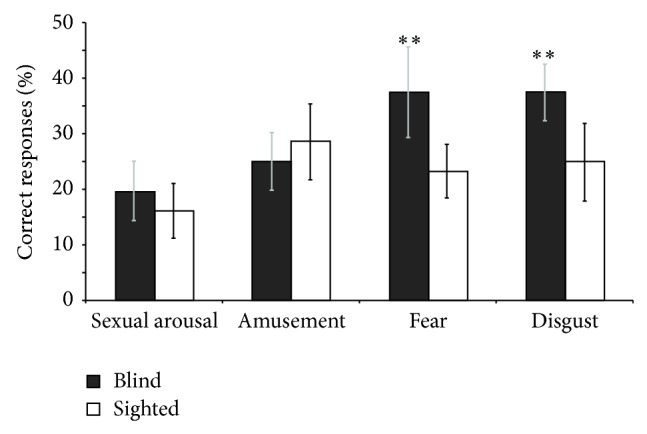
Results of the odour identification task. Congenitally blind participants had a strong tendency to be better at identifying fear and disgust, but none of the groups were above chance level (20%) for amusement and sexual arousal. Data present mean ± SD. ^**^ *P* < 0.01.

Figure 4 shows the results for the odour identification task. When asked to identify a particular emotion from the five jars, blind participants were above chance for disgust (mean accuracy = 38 ± 5%; *P* < 0.01) and fear (mean accuracy = 38 ± 8%; *P* < 0.006) but at chance level for amusement (mean accuracy = 20 ± 5%) and sexual arousal (25 ± 5%). In sharp contrast, sighted participants were at chance level for all four emotions. Mean accuracy for detecting disgust, sexual arousal, fear, and amusement was 25 ± 7%, 16 ± 5%, 23 ± 5%, and 29 ± 7%, respectively.

**Figure 5:**
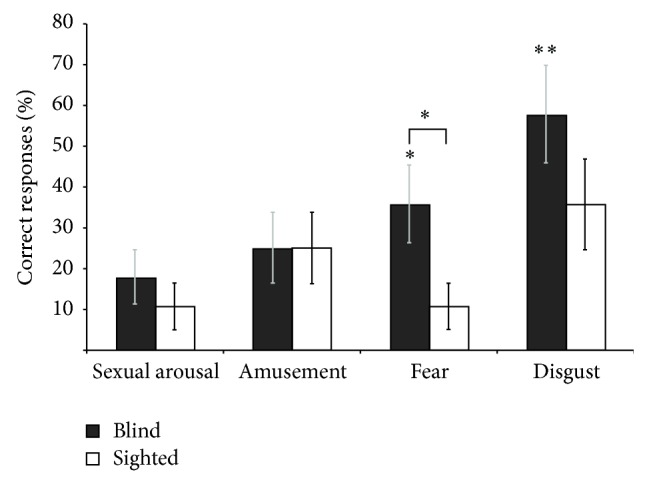
Results of the odour identification task of male sweat samples. Congenitally blind participants were significantly better than sighted controls at identifying fear from males and also had a strong tendency to be better at identifying disgust from males. None of the groups were above chance level (20%) for amusement or sexual arousal. Data presentmean ± SD. ^*^ *P* < 0.05; ^**^ *P* < 0.01.

The between group comparison revealed a strong tendency that the blind were better than the sighted controls at identifying fear (*z* = 1.36; *P* = 0.08) and disgust (*z* = 1.39; *P* = 0.08). When considering the results for the male sweat samples only (Figure 5), blind individuals were significantly better than sighted controls at identifying fear (*z* = 1.99; *P* = 0.02) and also had a tendency to be better at identifying disgust (*z* = 1.29; *P* = 0.097).

Finally, a comparison between males and females in odour identification did not reveal any significant group differences (*P* values > 0.26).

### 3.5. Odour Intensity Ratings

Sweat samples from male donors were rated as more intense than the emotional sweat samples from females (*t*(27) = 3.69, *P* < 0.001, *t*(27) = 3.5, *P* < 0.001, *t*(27) = 4, *P* < 0.001, and *t*(27) = 8.3, *P* < 0.001 for amusement, sexual arousal, fear, and disgust, resp.). There were no differences between males and females in body odour intensity ratings for any of the emotions (*P* values > 0.12).

## 4. Discussion

The aim of this study was to compare congenitally blind and sighted control individuals in their capacity at detecting emotions of a fellow human, conveyed through olfactory cues. Our results showed that congenitally blind subjects are better at identifying fear from male donors and that they have a strong tendency to be also better at identifying disgust.

In order to obtain a strong emotional response in the sweat donors, we used individualized film excerpts based on the donors' own film preferences. The high ratings on the questionnaire of emotional arousal that was filled in after the movie clips confirmed that we were indeed successful in inducing the intended emotions. This conclusion is supported by our hemodynamic measures that showed significant increases in heart rate during the film excerpts compared to baseline measurements. We can therefore conclude that the film excerpts were suitable to evoke the attended emotions in the donors.

It has previously been shown that congenitally blind subjects outperform matched controls in various olfactory tasks. For instance, congenitally blind subjects have a lower odour identification threshold [5] and perform better than matched sighted controls in free odour identification tasks and in olfactory tasks involving higher level cognitive process such as semantic memory for odours [20]. Here we show for the first time that congenitally blind subjects are also better at identifying emotional chemosensory cues in sweat samples of conspecifics. These results extend previous findings from our laboratory showing that congenitally blind individuals pay more attention to social body odours [5]. Together, the data suggest that blind subjects use body odours for purposes of social cognition. The improved ability to identify body odours may be explained by both intramodal and crossmodal plasticity. Evidence in favour of intramodal plasticity comes from Rombaux and coworkers, showing that the superior olfactory performance in congenitally blind subjects is associated with an increased volume of the olfactory bulb [21]. Data from our own laboratory show that congenitally blind individuals more strongly activate their primary and secondary olfactory areas during an odour identification task [14]. The results of this study further revealed strong occipital cortex activation during olfactory processing in the blind, hence suggesting that crossmodal neuroplastic processes could also contribute to the enhanced olfactory performance. Results from various human studies report that the olfactory system is indeed highly plastic [13] and that consequently it is susceptible to crossmodal changes similar to those observed for the tactile and auditory modalities [22].

Although our sighted controls were able to detect emotional body odours above chance level, they were not able to identify any of the emotions above chance level. This seems to be in contrast with previous results from Chen and Haviland-Jones who reported that humans are able to identify fear and amusement above chance level [23]. An explanation for this different result could be that Chen and Haviland Jones used a different paradigm paradigm, testing only two emotions, hence making the test easier. Furthermore, a more recent study from Zhou and Chen [24] failed to replicate these findings. A possible explanation why sighted individuals are able to detect but not to identify emotional body odours may reside in the fact that chemosensory signals are known to operate subtly and unconsciously [25–27], hence making it an extremely difficult task to identify the odours.

Congenitally blind were better at the identification of the negatively valenced emotions, but not of the positive ones. This is in line with animal data showing that mammalians use chemosignals particularly to communicate affective and emotional states of fear or distress. For instance, a recent study in rodents demonstrated a learned mother-to-infant transfer of fear to a novel odour [28]. There is a growing body of evidence that chemosensory signals of fear or anxiety can influence human behaviour and cognition unconsciously [6, 23, 26, 29–33]. For example, one study showed that chemosensory anxiety signals enhance the startle reflex, an indirect measure of anxiety [31]. An fMRI study reported amygdalar activation in response to fearful as compared to nonfearful body odours, despite the fact that participants were unable to distinguish between the two in a perceptual forced-choice paradigm [26]. Since the amygdala is involved in both emotional processing and olfactory processing [34], it is an obvious candidate in the chemosensory processing of emotions.

Interestingly, blind subjects were significantly better than their sighted controls at identifying body odour only from male donors. Studies of chemical analyses of sweat samples have shown that male signals of stress-related odours are stronger than those of females [35]. This could be due to the fact that the apocrine glands in the underarm are larger in men than in women [36], thereby producing a larger amount of chemosignalling molecules. This is confirmed by the higher intensity ratings of the male as compared to female sweat samples in our study.

Like fear, disgust is another evolutionary important emotion. Nonetheless, disgust has received only little attention in the field of chemosensory signalling. Disgust can serve to warn about contamination of food or noxious chemical stimulation, thereby enhancing survival [37]. The perception of disgust creates sensory rejection in order to reduce exposure [38]. For example, the facial expression of disgust when smelling something disgusting not only reduces the first person's sensory exposure to the smell but also warns conspecifics to stay away from the source that created the feeling of disgust. Although disgust is often communicated through visual input, there is some evidence that it can also be conveyed through body odours [11]. Our results show that disgust as a chemosensory signal becomes more important in the absence of vision.

In our study, none of the groups were above chance level for the detection of chemosensory signals of amusement. A study by Chen and Haviland-Jones also found a lack of ability to detect amusement as a chemosensory signal [23]. It may be argued that chemosensory signalling of amusement, which is an emotion generated in response to stimuli with socially acquired value, does not carry as much evolutionary salience as fearful sweat, which is generated in response to stimuli that threaten survival [39]. In our study, participants also failed to identify sexual body odours. This result is at odds with results of previous studies [40] and may be explained by methodological issues. Sexual arousal is more difficult to evoke in an experimental setting since it is potentially associated with feelings of embarrassment due to its intimate and private character. Even though the participants reported having been sexually aroused by the movie excerpts, sexually arousing sweat should have been collected in a more ecological condition, such as during sexual arousal of the sweat donor with his or her partner or from different body parts, for example, the groin region.

Though significant, the observed effects were somewhat subtle, which may not be surprising considering the relatively moderate sample sizes and the fact that the intensity and pleasantness of the emotional and sweat samples did not differ, preventing that these cues could help to distinguish between emotional and control odours.

## 5. Conclusions

In conclusion, our data indicate that congenitally blind people make a better use of olfaction when assessing other individual's emotional states. These findings suggest that when vision is lacking from birth, phylogenetically older forms of interacting with the world gain importance.

## Figures and Tables

**Table 1:** Demographic data of blind participants (M = male, F = female).

Sex	Age	Blindness onset	Blindness etiology	Residual vision
F	28	Birth	Retinopathy	None
M	29	Birth	Retinopathy of prematurity	None
F	32	Birth	Retinopathy of prematurity	None
M	39	Birth	Optic nerve atrophy	Light
M	43	Birth	Retinopathy of prematurity	Light
F	45	Birth	Retinopathy of prematurity	Light/shapes
M	46	12 months	Meningitis	Light/shapes
M	54	Birth	Retinopathy of prematurity	None
M	62	Birth	Retinopathy of prematurity	None
F	28	Birth	Retinopathy of prematurity	None
F	47	6 years	Leber's congenital amaurosis	None
M	45	Birth	Retinopathy of prematurity	None
F	53	Birth	Retinopathy of prematurity	None
F	65	Birth	Retinopathy of prematurity	None

**Table 2:** Heart rate increases during the movie clips.

Emotion	HR mean increase (SEM)	*t*-score	*P* value
Fear	27.5 ± 9.8	*t*(7) = 2.79	0.01
Disgust	21.6 ± 9.7	*t*(7) = 2.23	0.03
Amusement	32 ± 15.7	*t*(5) = 2.19	0.04
Sexual arousal	25.4 ± 16.2	*t*(6) = 1.57	0.08
